# Where do I submit my next paper? A practical guide for biomedical researchers

**DOI:** 10.7555/JBR.39.20250147

**Published:** 2026-05-21

**Authors:** Mohammad S. Alrashdan

**Affiliations:** 1 Department of Oral and Craniofacial Health Sciences, College of Dental Medicine, University of Sharjah, Sharjah, UAE; 2 Department of Oral Medicine and Oral Surgery, Faculty of Dentistry, Jordan University of Science and Technology, Irbid, Jordan

Dear Editor,

In addition to the classic barriers and challenges encountered during the design, conduct, and reporting of research, researchers and scholars now face a growing dilemma: selecting the most appropriate journal in which to publish their work. This decision requires careful consideration of the scope and quality of the submitted paper, as well as factors related to the target journal. This viewpoint briefly outlines the key factors that may influence this process and offers guidance to researchers on making an informed selection. A simplified flowchart summarizing the key steps in journal selection is provided in ***Fig. 1***.

**Figure 1:**
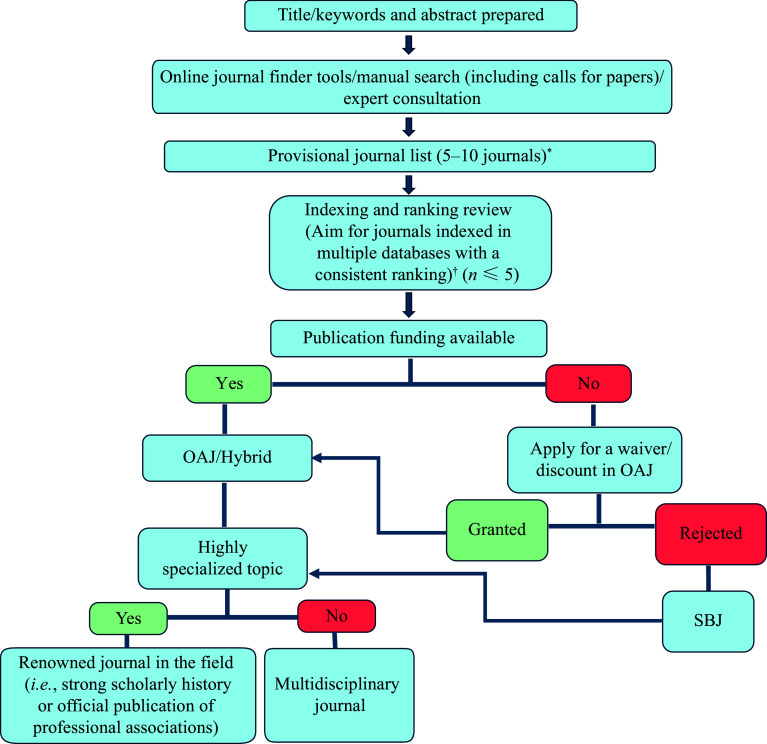
A simplified flowchart for journal selection. ^*^Apply predatory journal checks. ^†^Exceptions may apply in cases where new, non-indexed journals are considered, provided that they adhere to rigorous publication standards and editorial policies. Expert consultation is highly advised. Abbreviations: OAJ, open access journals; SBJ, subscription-based journals.

## The readership and scope of work

Defining the target readership of a particular article is a critical early step for authors to consider. While topics of broad interest, such as public health or preventive care, should be readily accessible to large audiences of both experts and non-experts, more specialized research that addresses a specific area of knowledge is better published in focused journals recognized by specialists in that field. This approach ensures that the paper reaches its intended readership, enhances its credibility, and reduces the risk of being overlooked among countless publications in non-specialized journals.

## Journal indexing and ranking

Journal reputation and ranking, closely tied to their impact factor (IF), are consistently acknowledged by authors as key factors in journal selection, primarily because of their implications for visibility and career advancement^[1–4]^. Gaston *et al*^[5]^ analyzed the annual submission data for all Wiley journals between 2007 and 2018 (more than one thousand journals) and reported a significant correlation between the number of submissions and a journal's IF, whereby journals received an average of 105 more submissions for every one-point increase in their IF. Another key indicator of a journal's reputation was the number of retractions, which demonstrated a correlation with lower submission rates.

Several journal indexing databases exist, implementing different metrics to rank journals, most of which are citation-based. While higher-ranking journals are generally perceived as more reliable sources of validated knowledge than lower-ranking ones, significant variations in the ranking of the same journal across different ranking systems are not uncommon. Therefore, a critical early step in the publishing process is determining which ranking system(s) to use as a reference.

One relevant consequence of routinely targeting top-ranking journals is the negative impact on younger journals, which often struggle to attract quality submissions until they become more visible and attractive to researchers, ultimately leading to improvements in their ranking, a process that usually takes several years.

## Open access or subscription model?

With more than 1.5 million new biomedical citations added to the PubMed database in 2023^[6]^, it is estimated that at least 60% of these are open access (OA)^[7]^. Furthermore, the reported annual growth rate in OA publications is around 30%^[8]^.

Papers published as OA have higher visibility and serve the ultimate goal of knowledge dissemination, making OA a generally favorable option for most authors^[9–10]^. However, a closer look at OA journals reveals that some are overly multidisciplinary and lack a well-defined target readership. Furthermore, the peer review process at some OA journals has been criticized for lacking adequate rigor, raising concerns about the quality of their published content^[8]^. Article processing charges (APC) also pose a significant constraint for researchers, particularly those with limited funding^[8]^. Nevertheless, many OA journals allow authors to apply for APC waivers or discounts at the submission stage. It is noteworthy that both OA and the less common model of free access journals provide unrestricted access to their content; however, they differ in the source of APC payment and copyright ownership, which is retained by the authors in OA journals but typically held by the publisher in free access journals.

Subscription-based (SB) journals, on the other hand, have minimal or no APC, and therefore provide a more convenient option for researchers with a limited budget; however, the visibility of any published work is significantly reduced. Some journals permit authors to share their preprints or accepted manuscripts on academic networking sites (*e.g.*, ResearchGate) upon request, offering an alternative means of access to publications, though this remains strictly subject to journal policies.

The third model used by some journals is the hybrid publishing model, whereby authors may choose to publish their work as OA for additional charges or as restricted access. Hybrid journals are generally perceived to maintain rigorous peer review standards since OA publishing and the associated APC are only applicable after the review process. Similar to SB journals, newly launched hybrid journals may initially struggle to build the momentum required to secure inclusion in scholarly databases and attain a competitive IF. In such cases, the reputation of the publisher is also likely to play a role.

## Policies set by academic or research institutions

Many academic and research institutions worldwide apply strict criteria for publication in scholarly journals and usually prefer those with a high ranking. These regulations are typically formalized and communicated to all scholars and faculty, and adherence to such regulations is closely monitored, especially when it comes to financial support or employee promotion. Most of these criteria pertain to journal inclusion and ranking in particular databases. While these practices are intended to benefit both the institutions and the researchers, they may redirect submissions toward a limited number of journals that fulfill the specified criteria, thereby hindering the growth and expansion of emerging ones.

## The review process and time constraints

Most journals now include average review times as part of their basic metrics, often highlighting shorter timeframes as a point of merit. Similarly, key publishers typically report review process metrics for their journals, such as the time from submission to first editorial decision and the time from submission to final acceptance, which may vary considerably. For example, in 2024, Nature Portfolio journals reported an average of 9 days from submission to first decision (range: 2–20 days), and an average of 188 days to acceptance (range: 103–345 days)^[11]^. While the influence of review timelines on journal selection is well documented^[1–2]^, it is generally considered less critical than other factors such as the IF or OA status^[5]^. One exception would be when researchers are facing institutional or funding-related time constraints, in which case they may prioritize a more reasonable turnaround time and a streamlined review process over a journal's reputation.

It is well recognized that, with the increasing volume of submissions received by journals, the review process has become more time-consuming and less predictable, with many papers taking more than 12 months to complete review process. These delays are often attributed to the difficulty of securing qualified reviewers and awaiting their feedback. However, this may be frustrating for authors and may even reduce the anticipated impact of the paper, as other papers on the same topic might be published in the interim. In this context, the timely communication of rejection decisions is just as important as that of acceptance decisions, as it prevents unnecessary delays and enables authors to seek alternative journals for their submissions.

## The artificial intelligence dilemma

Artificial intelligence (AI) is transforming scientific publishing, particularly in the biomedical field, by performing tasks such as manuscript editing, data analysis, and even peer review. Some journals permit authors to use AI tools, outlining specific guidelines in their author instructions, while others do not accept such use due to concerns over accuracy, transparency, and ethics.

A recent bibliometric analysis that included the top 100 academic publishers and scientific journals (regardless of subject area) found that 24% of publishers and 87% of journals provided guidance on the use of generative AI (GAI) tools^[12]^. The guidelines were generally similar among journals, including the top medical ones. Almost all publishers and journals with such guidelines (96% and 98%, respectively) did not approve of listing GAI as an author, while 8% of publishers and 22% of journals stated that their guidelines exclusively applied to the writing process. With regard to disclosure, 75% of publishers and 43% of journals requested that the use of GAI be disclosed, albeit in different sections of the manuscript.

The International Committee of Medical Journal Editors has recently recommended that authors disclose the use of AI in manuscript preparation, including details on which parts or stages were involved^[13]^. The statement disapproved of listing or citing AI or AI-assisted technologies as an author or co-author.

The application of AI in the peer review process is another emerging topic of interest, with preliminary data suggesting its potential usefulness in verifying manuscript adherence to guidelines, assisting in editorial tasks, and conducting initial screening of submitted papers^[14]^. However, concerns have been raised that the use of AI may compromise the confidentiality and integrity of the review process^[15]^.

Taken together, it is expected that AI will continue to contribute to scholarly content curation, development, and peer review, as well as to streamline the publication process while raising new challenges for validation and credibility^[14]^. There is a crucial need to establish standardized guidelines for the application of AI in scholarly publications, at least within individual subject areas.

In conclusion, careful planning for publication should be considered by researchers as early as possible, and the available options should be thoroughly assessed. Several factors influence journal selection, some of which are within the authors' control, such as the target readership and the perceived value and impact of the work, while others are dependent on the availability of publication funding and specific journal requirements.

## Examples

### Researcher A

This researcher is submitting a paper on a new diagnostic test for a rare kidney disorder. After preparing the title and abstract, and generating a provisional list of potential journals using journal finder tools and expert consultation, they proceed through the following steps: (1) Their topic is highly specialized, and the target audience is limited (primarily clinical nephrologists or diagnostic scientists); (2) The researcher has no significant constraint regarding publication funding; and (3) Therefore, they are likely to select a highly focused, subscription-based journal with a strong reputation in the field (*e.g.*, an official journal of a nephrology association).

### Researcher B

This researcher has conducted a population-based study on the relationship between lifestyle factors and cognitive function in adults. After generating a list of candidate journals as described above, they proceed as follows: (1) The study has broader public health implications and may interest a wide and interdisciplinary audience; (2) The author should ideally secure adequate publication funding; if not, they should consider applying for APC waivers or discounts in an OA journal; and (3) Since the topic is not highly specialized, a multidisciplinary OA journal with a broad readership across public health and related sciences would be an appropriate target.

Yours sincerely,Mohammad S. Alrashdan^1,2,^^✉^^1^Department of Oral and Craniofacial Health Sciences, College of Dental Medicine, University of Sharjah,Sharjah, United Arab Emirates;^2^Department of Oral Medicine and Oral Surgery, Faculty of Dentistry, Jordan University of Science and Technology, Irbid 22110, Jordan.^✉^Corresponding author: Mohammad S. Alrashdan, E-mail: malrashdan@sharjah.ac.ae, ORCID: 0000-0003-2512-1557.
